# Implicit sensorimotor adaptation is preserved in Parkinson’s disease

**DOI:** 10.1093/braincomms/fcac303

**Published:** 2022-11-21

**Authors:** Jonathan S Tsay, Tara Najafi, Lauren Schuck, Tianhe Wang, Richard B Ivry

**Affiliations:** Department of Psychology, University of California Berkeley, Berkeley, CA 94704, USA; Helen Wills Neuroscience Institute, University of California Berkeley, Berkeley, CA 94704, USA; Department of Psychology, University of California Berkeley, Berkeley, CA 94704, USA; Department of Psychology, University of California Berkeley, Berkeley, CA 94704, USA; Department of Psychology, University of California Berkeley, Berkeley, CA 94704, USA; Department of Psychology, University of California Berkeley, Berkeley, CA 94704, USA; Helen Wills Neuroscience Institute, University of California Berkeley, Berkeley, CA 94704, USA

**Keywords:** motor learning, visuomotor adaptation, Parkinson’s disease, error-based learning

## Abstract

Our ability to enact successful goal-directed actions involves multiple learning processes. Among these processes, implicit motor adaptation ensures that the sensorimotor system remains finely tuned in response to changes in the body and environment. Whether Parkinson’s disease impacts implicit motor adaptation remains a contentious area of research: whereas multiple reports show impaired performance in this population, many others show intact performance. While there is a range of methodological differences across studies, one critical issue is that performance in many of the studies may reflect a combination of implicit adaptation and strategic re-aiming. Here, we revisited this controversy using a visuomotor task designed to isolate implicit adaptation. In two experiments, we found that adaptation in response to a wide range of visual perturbations was similar in Parkinson’s disease and matched control participants. Moreover, in a meta-analysis of previously published and unpublished work, we found that the mean effect size contrasting Parkinson’s disease and controls across 16 experiments involving over 200 participants was not significant. Together, these analyses indicate that implicit adaptation is preserved in Parkinson’s disease, offering a fresh perspective on the role of the basal ganglia in sensorimotor learning.

## Teaser

Tsay *et al*. examined the effect of Parkinson’s disease on sensorimotor adaptation. The results from two experiments and a meta-analysis provide compelling evidence that implicit adaptation is preserved in participants with Parkinson’s disease, and suggest that the contribution of the basal ganglia on adaptation tasks may be related to other learning processes.

## Graphical Abstract

**Graphical Abstract:**
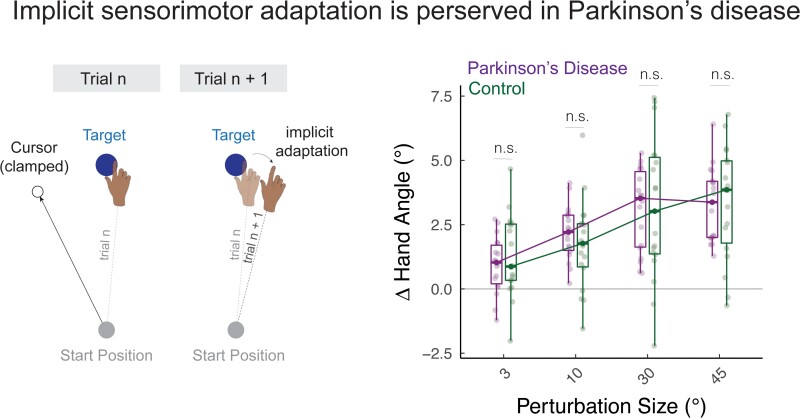

## Introduction

Parkinson’s disease, a neurodegenerative disorder primarily affecting the basal ganglia circuitry, is thought to impair the ability to acquire and adapt skilled movements.^1–4^ Evidence of this comes from research involving a wide range of motor learning tasks including sequence learning,^5–9^ sensorimotor adaptation^10,11^ and skill acquisition.^12^ Coupled with evidence implicating the basal ganglia in habitual behaviour,^13,14^ this body of work has motivated the idea that the basal ganglia is essential for the acquisition, refinement and automatization of skilled movement.^15^

**Table 1:** Parkinson's disease and matched control participants

Exp	Group	*N*	Age	Gender	Handedness	Years of Education	MoCA	UPDRS
1	Parkinson's disease	18	65.7 (7.6)	8 M, 10 F	14R, 3L, 1A	17.4 (3.3)	27.8 (1.8)	16.6 (5.1)
	Control	18	64.1 (6.9)	8 M, 10 F	14R, 3L, 1A	15.3 (2.2)	–	–
2	Parkinson's disease	16	66.9 (7.2)	7 M, 9 F	13R, 3L	17.6 (3.2)	27.4 (1.9)	16.7 (5.0)
	Control	16	65.6 (7.2)	7 M, 9 F	13R, 3L	15.4 (2.6)	–	–

Participants reported either being right-handed (R), left-handed (L) or ambidextrous (A). With our modified test, UPDRS scores can range from 0 to 40 (where high numbers indicate greatest symptomology) and MoCA scores can range from 0 to 30 (where low numbers indicate greatest impairment). Mean (SD) is provided.

In the present study, we focus on one form of sensorimotor learning, visuomotor adaptation. This type of learning keeps the motor system exquisitely calibrated by automatically adjusting the sensorimotor map in response to errors between the expected and actual sensory feedback.^16,17^ A number of studies have shown that individuals with Parkinson's disease are impaired on visuomotor adaptation tasks^18–20^; specifically, when a perturbation is imposed on the visual feedback, participants with Parkinson's disease fail to show the same level of compensation (i.e. movement in the opposite direction of the perturbation) as control participants. However, recent studies have revealed the operation of multiple learning processes even in seemingly simple tasks such as visuomotor adaptation. In particular, implicit adaptation may be supplemented by the use of volitional aiming. For example, to nullify an angular perturbation of the visual feedback, participants may aim away from the target in a strategic manner.^21,22^ As such, the impaired performance of Parkinson's disease participants on adaptation tasks may not reflect disruption to implicit adaptation per se, but an impairment in other learning processes such as strategic aiming (see also^23^).

To address this concern, we assessed Parkinson's disease and matched controls in a visuomotor adaptation task that isolates implicit motor adaptation.^24^ In this task, participants reach to a visual target and receive cursor feedback that follows a trajectory defined relative to the target and, importantly, is not contingent on the trajectory of the participant’s actual movement. Participants are fully informed of this manipulation and instructed to always reach directly to the target while ignoring the visual feedback. Despite these instructions, the mismatch introduced between the position of the target and the visual cursor induces an automatic adaptive response in healthy young^24–29^ and older^30^ participants, causing a drift in movement direction away from the target and in the opposite direction of the cursor. These motor corrections are not the result of re-aiming; indeed, participants are oblivious to their change in their behaviour.^31,32^

We tested individuals with Parkinson's disease in two web-based experiments with this form of non-contingent feedback (for a validation of our web-based platform, see^26,33^). We complemented our empirical work with a meta-analysis, reviewing the results from 16 visuomotor adaptation experiments that allowed for a comparison between Parkinson's disease and control groups on an aftereffect measure *presumed* to index the implicit processes underlying motor adaptation (but in our Discussion section, we will outline why aftereffects may also be a contaminated measure). If the basal ganglia are involved in implicit adaptation, we expect that Parkinson's disease participants will exhibit attenuated trial-by-trial implicit motor corrections in response to a wide range of visual perturbations (Exp 1), attenuated accumulated learning in response to a consistent visual error (Exp 2), and exhibit an overall pattern of impairment in our meta-analysis of published and unpublished work. Taken together, we sought to provide a thorough examination of the impact of Parkinson's disease on implicit motor adaptation.

## Materials and methods

### Ethics statement

All participants gave written informed consent in accordance with policies approved by UC Berkeley’s Institutional Review Board (protocol number: 2016-02-8439). Participation in the study was conducted online and in exchange for monetary compensation.

### Participants

Individuals diagnosed with Parkinson’s disease were recruited via our clinical database. The database is composed of individuals from the USA who have responded to online advertisements distributed by leaders of local support groups. We follow-up with a video call to describe the project in detail. For those who wish to participate, we obtain a medical history, neurological evaluation of Parkinson's disease symptoms using the motor component of the unified Parkinson’s disease rating scale (UPDRS),^34^ and evaluation of general cognitive status with the Montreal cognitive assessment (MoCA).^35,36^ The UPDRS and MoCA were modified for online administration.^33^ For the UPDRS, we eliminated one item (postural stability), and three items were scored based on self-reports (arising from chair, posture and gait) due to concerns about safety during remote examination. For the MoCA, we eliminated the Alternating Trail Making item since it requires a paper copy. As shown in Table 1 and Supplementary Table S1, Parkinson's disease participants tended to have mild (0–20) to moderate (21–40) motor impairments. Four of Parkinson's disease participants exhibited mild cognitive impairment (MCI) (MoCA score <26). Note that these Parkinson's disease participants with mild MCI were not excluded for two reasons: (i) *A priori*, we do not expect that implicit adaptation is modulated by cognitive ability; (ii) despite being below the typical cut-off for MCI, these individuals scored well above the cut-off for Parkinson’s dementia.^37^

A total of 25 unique individuals with Parkinson's disease participated in our experiments. Eighteen were tested in Experiment 1 and 16 in Experiment 2, with nine participants completing both experiments. The two experiments were separated by at least 6 months. Parkinson's disease participants were on their normal medication schedule at the time of the video call and online testing. Our sample size was based on the average sample size recruited in previous studies examining the effect of Parkinson's disease on motor adaptation (average number of participants in studies included in our Parkinson's disease meta-analysis was 16). We conducted a *post hoc* power analysis (alpha = 0.05; power = 0.95; two-tailed independent-sample *t*-tests) using Cohen’s *D* from the three studies that have found group differences in implicit adaptation between Parkinson's disease and control participants (Contreras-Vidal *et al*.: *D* = 2.81; Singh *et al*.: *D* = 1.38; and Venkatakrishnan *et al*.: *D* = 1.33). This analysis suggested a minimal sample size of 4, 10 and 11, respectively. As such, the sample sizes in both experiments had sufficient power to detect group differences.

Control participants were recruited via Prolific, a website for online participant recruitment. We created a prolific advertisement for each control participant to match each Parkinson's disease participant based on gender, self-reported handedness and age (±5 years old), resulting in sample sizes of 18 and 16 controls for Experiments 1 and 2, respectively. Each control participant was tested in a single experiment. Parkinson's disease group had more years of education than the control group [Exp 1: *t*_30_ = 2.1, *P* = 0.04, (0.06, 4.3), *D* = 0.7; Exp 2: *t*_34_ = 2.3, *P* = 0.02, (0.2, 4.0), *D* = 0.8]. We did not include years of education in our selection criteria given that implicit motor adaptation is unlikely to depend on this measure. Nonetheless, given this group difference, we examined the possible impact of years of education as a covariate of interest in both experiments.

### Apparatus and procedure

Participants used their own laptop or desktop computer to access a customized webpage that hosted the experiment.^26,38,39^ Participants used their computer trackpad or mouse to perform the reaching task (sampling rate typically ∼60 Hz). The size and position of stimuli were scaled based on each participant’s screen size. For ease of exposition, the stimuli parameters reported below are for a typical monitor size of 13″ with a screen resolution of 1366 × 768 pixels.^40^

Reaching movements were performed by using the computer trackpad or mouse to move the cursor across the monitor. Each trial involved a planar movement from the centre of the workspace to a visual target. The centre position was indicated by a white circle, and the target location was indicated by a blue circle (both 0.5 cm in diameter). On the typical monitor, the radial distance from the start location to the target was 6 cm. The target appeared at one of two locations on an invisible virtual circle (135° = upper left quadrant; 315° = lower right quadrant). The movement involved some combination of joint rotations about the arm, wrist and/or finger depending on whether the trackpad or mouse was used. In our prior validation work using this online interface and procedure, the exact movement and the exact device used did not impact measures of performance or learning on visuomotor adaptation tasks.^26^ Previous work using the clamped feedback task^28^ indicates that implicit adaptation does not vary across target locations.

To initiate each trial, the participant moved the cursor, represented by a white dot (0.5 cm in diameter) into the start location. Feedback during this initialization phase was only provided when the cursor was within 2 cm of the start circle. Once the participant maintained the cursor in the start position for 500 ms, the target appeared. The participant was instructed to reach, attempting to rapidly ‘slice’ through the target. We did not impose any constraints on reaction time. However, to discourage mid-movement corrections, we required that the movement be completed within 500 ms. If the movement time exceeded this criterion, the message ‘Too Slow’ was displayed in the centre of the screen for 750 ms.

**Figure 1:**
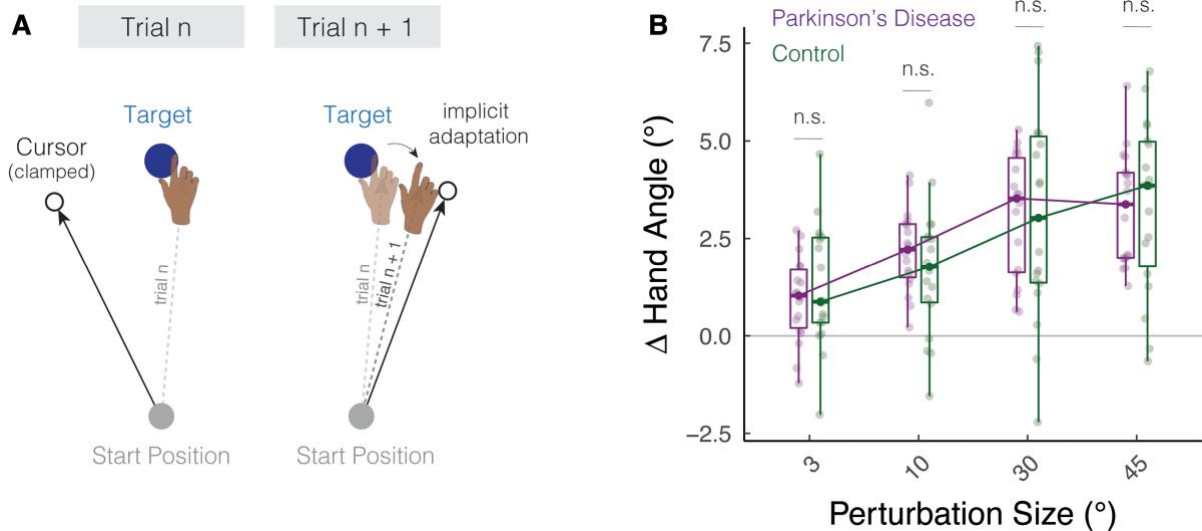
**Parkinson’s disease does not impact implicit adaptation in response to a wide range of error sizes.** (**A**) Schematic of the task. The cursor feedback (hollow black circle) was rotated relative to the target, independent of the position of the participant’s hand. The size of the rotation was varied randomly on a trial-by-trial basis. (**B**) Average change in hand angle on the subsequent trial is plotted as a function of the rotation size for Parkinson's disease and control participants. Box plots denote the median (thick horizontal lines), quartiles (first and third, the edges of the boxes) and extrema (min and max, vertical thin lines). The individual means are shown as translucent circles. n.s. denotes that the group comparison between Parkinson's disease and controls is not significant.

**Figure 2:**
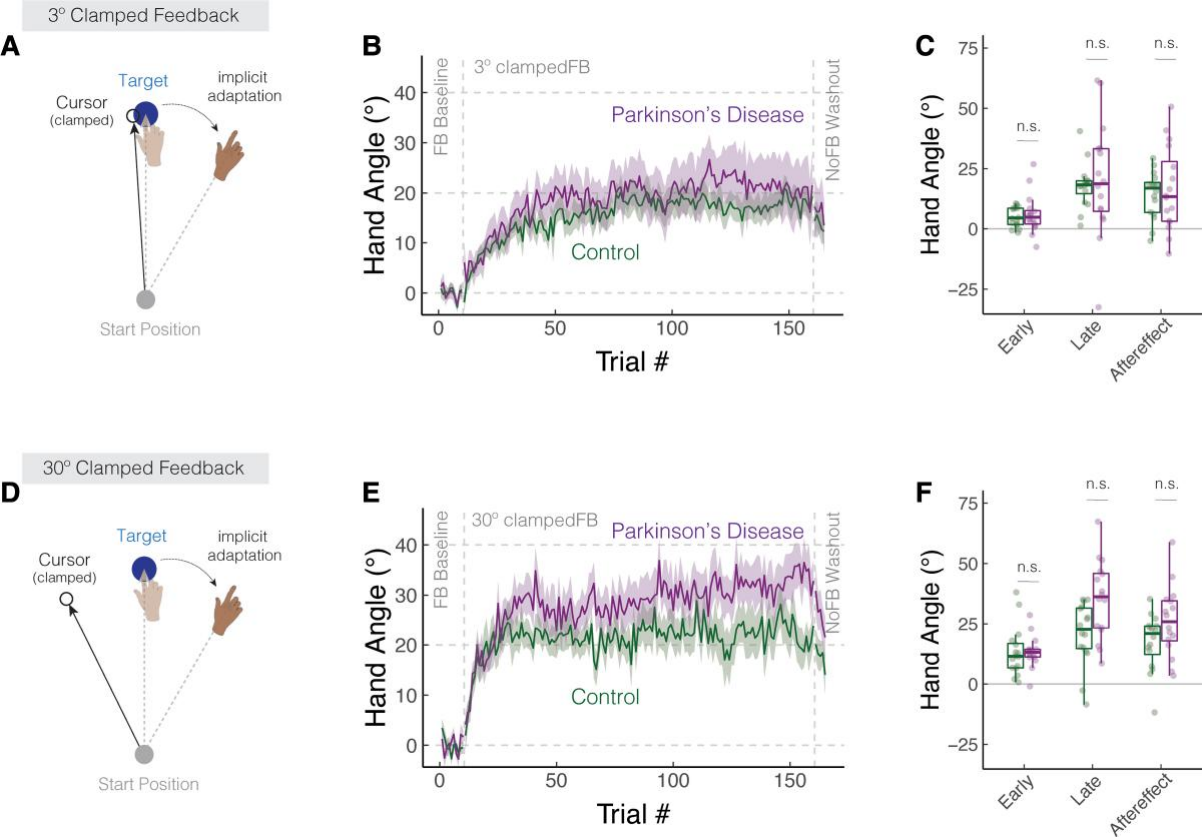
**Parkinson’s disease does not impact learning functions in response to clamped feedback.** (**A**,**D**) Schematic of the clamped feedback task. The cursor feedback (hollow black circle) followed an invariant trajectory, rotated by either 3° (**A**) or 30° (**D**) relative to the target. The rotation size remained invariant over a block of 110 trials, with the order and sign of the rotation size counterbalanced across participants. Participants were instructed to always move directly to the target (blue circle) and ignore the visual feedback. The translucent and solid colours display hand position early and late in adaptation, respectively. (**B**,**E**) Mean time courses of hand angle for the 3 and 30° conditions for Parkinson's disease and control participants. Data for each participant were baseline subtracted relative to mean hand angle during the baseline phase with veridical feedback. Shaded region denotes SEM. (**C**,**F**) Average hand angles during early and late phases of the perturbation block, and during the no-feedback aftereffect block. Box plots denote the median (thick horizontal lines), quartiles (first and third, the edges of the boxes) and extrema (min and max, vertical thin lines). The mean for each participant is shown as translucent circles. n.s. denotes that the group comparison between Parkinson's disease and Controls is not significant.

We assumed that the stimulus display (vertical) and movement (horizontal) were in orthogonal planes. Given this assumption, the feedback cursor during the centre-out movement could take one of three forms: congruent feedback, rotated non-contingent feedback and no-feedback. During congruent feedback trials, the movement of the visual feedback was congruent with the direction of the hand (e.g. rightward movement of hand produced a rightward movement of cursor; forward movement of hand produced an upward movement of cursor). During rotated non-contingent feedback trials (Fig. 1A, Fig. 2A and 2D), the cursor moved at a specified angular offset relative to the position of the target, regardless of the movement direction of the hand. The radial position of the cursor corresponded to that of the hand up to 6 cm, at which point the cursor position was frozen for 50 ms before disappearing. During no-feedback trials, the feedback cursor was extinguished as soon as the hand left the start circle and remained off for the entire reach. During the return phase after the movement, the (congruent) cursor was provided when the participant moved within 2 cm of the start circle.

### Experiment 1: the impact of Parkinson's disease on implicit adaptation

Parkinson's disease and control participants (*N* = 18 per group) completed an adaptation task in which we examined the trial-by-trial response to non-contingent feedback.^41,42^ To familiarize participants with the apparatus and task requirements, the experiment began with a short baseline phase (10 trials) with congruent feedback. This was followed by 220 trials with non-contingent feedback in which the angular offset of the feedback cursor from the target varied from trial to trial, both in direction (clockwise − or counterclockwise +) and magnitude (3, 10, 30 and 45${}^{\circ }$). There were 26–28 trials per perturbation size provided in a random, zero-mean order (i.e. every perturbation was repeated at least once every eight trials) to prevent any systematic drifts in hand angle too far away from the target.

Testing at the two target locations was varied across blocks, with one location used for trials 1–120 and the other location for trials 121–230 (i.e. each half contained 10 baseline congruent feedback trials and 110 non-contingent feedback trials). The target order was counterbalanced across participants. (We included the two locations to maintain a similar procedure in Experiments 1 and 2—see *Experiment 2* methods below.)

Prior to the start of each baseline block, the instruction ‘Move directly to the target as fast and accurately as you can’ appeared on the screen. Prior to the start of each perturbation block, the instructions were modified to read: ‘The white cursor will no longer be under your control. Please ignore the white cursor and continue to aim directly towards the target.’ To clarify the invariant nature of the clamped feedback, three demonstration trials were provided before the first perturbation block. On all three trials, the target appeared straight ahead (90° position), and the participant was told to reach to the left (Demo 1), to the right (Demo 2) and backward (Demo 3). On all three of these demonstration trials, the cursor moved in a straight line, 90° offset from the target. In this way, the participant could see that the spatial trajectory of the cursor was unrelated to their own reach direction.

Hand angle was defined as the position of the hand relative to the target when movement amplitude reached 6 cm. Owing to data storage constraints, we opted not to record the entire movement trajectory since pilot data indicated that movement trajectories were relatively straight with minimal mid-movement corrections. As our key measure of trial-by-trial adaptation, we calculated the change in hand angle on trial *n* + 1 as a function of the clamped rotation size on trial *n*. The mean trial-by-trial change in hand angle was calculated for each perturbation size (combing both clockwise and counterclockwise directions).

The mean data were submitted to a linear mixed effect model (R statistical package: lmer), with rotation size (3, 10, 30 and 45°) and group (Parkinson's disease or control) as fixed effects and participant ID as a random effect. We also included Years of Education as a covariate, since this factor differed between groups. To visualize the data, the change in hand angle values with counterclockwise rotations were flipped, such that a positive hand angle corresponds to an angle in the opposite direction of the rotated feedback, the direction of movement expected from implicit adaptation.

### Experiment 2: the impact of Parkinson's disease on the upper bound of implicit adaptation

As a second test of the effect of Parkinson's disease on implicit adaptation, we measured the cumulative effects of learning, an approach that should magnify any subtle differences between groups. To test this, we used a clamp of a fixed size and direction throughout the perturbation block, allowing learning to accumulate in one direction (i.e. the opposite direction of the perturbation). With this method, our focus was on the upper bound of adaptation and the aftereffect.

Parkinson's disease and control participants (*N* = 16 per group) completed this task. To familiarize participants with the apparatus and task requirements, the experiment began with a short baseline block (10 trials) with congruent feedback. This was followed by a clamped non-contingent feedback perturbation block for 150 trials and a no-feedback aftereffect block for five trials. All three phases were then repeated at a different target location, amounting to a total of 330 trials

As in Experiment 1, the target again appeared in one of two locations (135°, upper left quadrant; 315°, lower right quadrant). One target was paired with a small (3°) perturbation, and the other was paired with a large (30°) perturbation. The targets were spaced apart by 180° to eliminate generalization of learning between targets; generalization of implicit adaptation is minimal for targets spaced more than 45° apart.^24,43,44^ The pairings between targets and clamp sizes were counterbalanced across individuals. We also counterbalanced the presentation of the clamp sizes to nullify potential session order effects.^27^ Together, our approach yielded eight unique combinations (two target locations × two clamp sizes × two test orders).

Task instructions were similar to that of Experiment 1: Prior to the baseline block, the participant was instructed to ‘Move directly to the target as fast and accurately as you can.’ Prior to the non-contingent clamp block, the instructions were modified to read: ‘The white cursor will no longer be under your control. Please ignore the white cursor and continue to aim directly towards the target.’ We again included three demonstration trials prior to the first clamped perturbation block, with the procedure identical to that of Experiment 1. Prior to the no-feedback aftereffect block, participants were again instructed to ‘Move directly to the target as fast and accurately as you can.’

To evaluate the magnitude of adaptation, the hand angle for each trial was calculated with respect to the participant’s idiosyncratic baseline bias.^45,46^ Baseline was defined as the mean hand angle during the 10 trials of the baseline block, movements that had been performed with congruent feedback (first target: trials 1–10; second target: trials 166–175). We defined three measures of learning: early adaptation, late adaptation and aftereffect. Early adaptation was operationalized as the mean hand angle over the first 10 trials of the perturbation block (first target: trials 11–20; second target: 176–185). Late adaptation was defined as the mean hand angle over the last 10 trials of the perturbation block (first target: trials: 151–160; second target: 316–325). The aftereffect was operationalized as the mean hand angle over the five trials of the no-feedback aftereffect block (first target: trials 161–165; second target: trials 326–330).

For each dependent variable, the data were submitted to a linear mixed effect model. We included rotation size (3 and 30°) and group (Parkinson's disease or control) as fixed effects and participant ID as a random effect. As in Experiment 1, Years of Education was included as a covariate.

### Statistical analysis

In Experiment 1, outlier trials were defined as (i) trials in which the change in hand angle on trial *n* + 1 differed by more than 3 SD from the mean change in hand angle (calculated separately for each clamp size), or (ii) trials in which the change in hand angle exceeded 75°. This resulted in 4.5 ± 3.3% (median ± IQR) trials removed from the controls dataset and 5.5 ± 6.5% removed from Parkinson's disease dataset. In Experiment 2, outlier trials were defined as trials in which the hand angle differed by more than 3 SD from a five-trial moving average (all trials included). This resulted in 1.0 ± 0.5% (median ± IQR) trials removed from the control dataset and 1.2 ± 0.6% removed from Parkinson's disease dataset. This percentage is higher than in typical in-person studies, a pattern that has been observed previously in online studies with young adults.^26^

In both experiments, we asked whether the learning measures for Parkinson's disease group were correlated with motor symptom severity (UPDRS) and/or cognitive status (MoCA) (R function: cor.test). These variables were not included as covariates in the linear mixed effect model since they were only assessed in Parkinson's disease group.

We employed F-tests with the Satterthwaite method to evaluate whether the coefficients (i.e. beta values) obtained from the linear mixed effects model were significant (R function: anova). Pairwise *post hoc t*-tests (two-tailed) were used to compare the learning measures between Parkinson's disease and control groups (R function: emmeans). *P*-values were adjusted for multiple comparisons using the Tukey method, and 95% confidence intervals are reported in squared brackets. Standard effect size measures were also provided (*D* for between-participant comparisons; *D*_*z*_ for within-participant comparisons; $\eta_{p}^{2}$ for between-subjects ANOVA).^47^ We evaluated key null results using a Bayes Factor, BF_01_ (R package: BayesFactor).

## Results

### Experiment 1: the impact of Parkinson's disease on implicit adaptation

We asked how Parkinson’s disease impacts implicit adaptation in response to a wide range of error sizes. To address this question, we varied the perturbation direction and the size of the non-contingent visual feedback using a random perturbation schedule throughout the perturbation block (Fig. 1A). We assayed implicit adaptation by measuring the change in hand angle from trial *n* to trial *n* + 1 as a function of the rotation size on trial *n*. Positive values indicate a change in hand angle opposite to the direction of the visual feedback (i.e. an implicit adaptive response). The size (3, 10, 30 and 45°) and direction (clockwise and counterclockwise) of the visual perturbations were programmed pseudo-randomly such that the mean perturbation was zero every eight trials. This allowed us to efficiently measure adaptation across a wide range of visual errors with the participant’s hand remaining close to the target.

As shown in Fig. 1B, Parkinson's disease (green) and control (dark magenta) participants exhibited an adaptive response, with the mean trial-to-trial change in hand angle in the opposite direction of the perturbation. This pattern was observed for all rotation sizes. Statistically, we first confirmed that the change in hand angle for each rotation size was significantly different than zero [all $t_{35}>4.9,$$p\left\langle 0.01;\;D\right\rangle 0.8;\;3^{\circ }:\;0.1,(0.6,1.6);\;10^{\circ }:\;0.9,(1.5,2.4);30^{\circ }:$$3.1,(2.3,3.9);\;45^{\circ }:\;3.3,\;(2.7,3.9)$]. Moreover, the magnitude of these trial-to-trial motor corrections converged with those observed in previous in-lab^30,42,48^ and online studies^26^ of implicit adaptation.

Implicit trial-to-trial motor corrections are known to increase with perturbation size only within a limited range, saturating in response to larger perturbations.^26,41,49–55^ This sublinear ‘motor correction’ function is thought to reflect an upper bound to trial-by-trial plasticity in either the sensory^56^ or motor system.^28^ Visual inspection of the data (Fig. 1B) indicated that the shape of the motor correction function was sublinear, increasing from 0 to 30° but saturating between 30 and 45°. Statistically, we verified this phenomenon in two ways: First, there was a main effect of rotation size [$F_{1106}=\;48.8,\;P<0.001,\;\beta =\;0.05,$$(0.03,0.07),\;\eta_{P}^{2}=0.3$], with the magnitude of the adaptive response increasing with the size of the perturbation. Second, the slope values computed with all rotation sizes were smaller than the slope values computed only with rotation sizes in the visually defined linear zone (3, 10 and 30°), indicating that the motor correction functions were sublinear [*t*_35_ = 2.3, *P* = 0.03, *β* = 0.02, (0.0, 0.04), *D*_*z*_ = 0.4]. We obtained a similar pattern of results in a secondary analysis in which Rotation Size was treated as a categorical variable (see Supplementary Results: Analysing rotation size as a categorical variable).

Turning to our main question, we next asked whether implicit adaptation would be impacted by Parkinson's disease. The main effect of group [$F_{1,69.2}=\;0.0,P=0.89,\beta =\;-0.01,$$(-1.2,1.0),\;\eta_{P}^{2}=0.0,\;BF_{01}=0.2$] and the group × rotation size $[F_{1106}=\;0.0,\;P=0.93,\;\beta =\;0.0,(-0.03,\;0.03),$$\eta_{P}^{2}=0.0,BF_{01}=0.2$] were not significant. A *post hoc* comparison of the slopes showed a similar null effect, with a Bayes factor providing evidence in favour of the null hypothesis (*t*_34_ = − 0.1, *P* = 0.93, ( − 0.04, 0.03), *D* = 0.0, *BF*_01_ = 0.3]. In summary, the results of Experiment 1 indicate that implicit adaptation is relatively preserved in Parkinson's disease across a wide range of rotation sizes.

### Experiment 2: the impact of Parkinson's disease on the upper bound of implicit adaptation

Experiment 2 provided a second test of the effect of Parkinson's disease on implicit adaptation. To measure the time course of learning, we used clamped non-contingent feedback with the size of the clamp set to a constant 3 or 30° in separate phases of the experiment (Fig. 2A and D). In this manner, we obtain a picture of the cumulative effects of learning, an approach that should magnify any subtle differences between groups. In previous studies with this method, participants exhibited a robust change in hand angle in the opposite direction of the cursor, with the asymptotic value typically ranging between 15 and 25°. This asymptote has been modelled to reflect the point where learning and forgetting offset one another,^57–60^ the upper bound on plasticity in the sensorimotor map^28^ or the point where multisensory integration is optimized.^56^

During the perturbation block, there was a gradual change in hand angle in the opposite direction of the clamped feedback, with the group-averaged functions approaching an asymptotic level after 50–100 clamped trials (Fig. 2B and E). The adapted response was largely maintained during the following no-feedback block, consistent with what would be expected if learning induced by the clamped feedback is implicit and automatic.

Both groups exhibited robust changes in hand angle in all three phases of the experiment [*t*-test against 0: early adaptation, Parkinson's disease: *t*_31_ = 6.7, *P* < 0.001, (6.8, 12.7); controls: *t*_31_ = 5.8, *P* < 0.001, (5.9, 12.3); late adaptation, Parkinson's disease: *t*_31_ = 7.3, *P* < 0.001, (20.0, 35.4); controls: *t*_31_ = 9.8, *P* < 0.001, (15.4, 23.4); aftereffect, Parkinson's disease: *t*_31_ = 7.3, *P* < 0.001, (15.3, 27.2); controls: *t*_31_ = 8.4, *P* < 0.001, (11.9, 19.7)]. Implicit adaptation scaled with the size of the rotation during early adaptation [main effect of rotation size: $F_{1,30}=\;20.1,P<0.001,$$\beta =\;0.3,\;(0.1,\;0.5),\;\eta_{P}^{2}=0.4$], but reached similar values for the 3° and 30° clamps in late adaptation [$F_{1,30}=0.7,\;P=0.51,\;\beta =\;0.1,\;(-0.2,\;0.4),\;\eta_{P}^{2}=0.2$] and in the aftereffect phase [$F_{1,30}=1.0,\;P=0.33,\;$$\beta =\;0.1,\;(-0.1,0.4),\;\eta_{P}^{2}=0.2$]. This asymptotic convergence is similar to that reported in Kim *et al*.^28^ (but see^61,62^; also Supplementary Discussion: The convergence of small and large visual errors in implicit sensorimotor adaptation; also see, Supplementary Table 2).

The learning functions for Parkinson's disease and control groups were statistically similar across all phases of the experiment, a null pattern that was observed in response to both the 3 and 30° clamps (Fig. 2C and F). The main effect of group and the group × rotation size interaction were not significant for all three dependent variables [main effect of group, early: $F_{1,58}=\;0.1\;P=0.76,\;\beta =\;1.0,\;(-4.9,\;6.9),$$\eta_{P}^{2}=0.0,\;BF_{01}=0.3$; late: $F_{1,53}=\;0.2,\;P=0.70,\;\beta =\;2.5,(-9.9,\;14.9),\;$$\eta_{P}^{2}=0.0,\;BF_{01}=2.0$; aftereffect: *F*_1,50_ = 0.4, *P* = 0.51, *β* = 3.6, (− 6.6, 13.7), $\eta_{P}^{2}=0.0,BF_{01}=1.8$; group × rotation size interaction, early: $F_{1,30}=\;0.1,\;P=0.78,\beta =\;0.0,$$(-0.3,\;0.2),\;\eta_{P}^{2}=0.0,\;BF_{01}=1.0$; late: *F*_1,30_ = 1.9, *P* = 0.07, *β* = 0.4, (0.0, 0.9), $\eta_{P}^{2}=0.1,\;BF_{01}=0.5$; aftereffect: $F_{1,30}=\;1.8,\;P=0.19,$$\hat{a}=0.2,\;(-0.1,0.6),\;\eta_{P}^{2}=0.1,\;BF_{01}=0.3$]. Moreover, these null results were robust at both target locations (see Supplementary Figure 1). If anything, there was a trend towards greater implicit adaptation in Parkinson's disease group. The underlying reason for this trend is unclear, perhaps driven by a few ‘super adapters’ (i.e. individuals with a heading angle that deviated by more than 25° from the target location during late adaptation). Importantly, our meta-analysis of the literature (see next section) did not provide any evidence of enhanced adaptation in Parkinson's disease.

In summary, the results of Experiment 2 converge with those obtained in Experiment 1, providing additional evidence that implicit adaptation to a visuomotor perturbation is preserved in Parkinson's disease for both small and large error sizes.

### Meta-analysis of the effect of Parkinson's disease on visuomotor adaptation

We conducted a meta-analysis of previous studies examining the effect of Parkinson's disease on visuomotor adaptation (see Supplementary Methods: Meta-analysis of the Effect of Parkinson's disease on Visuomotor Adaptation). Most of these studies used a standard perturbation, one in which the position of the feedback is contingent on the participant’s hand position. As such, adaptation results in improved performance with the cursor landing closer to the target. Recent work has shown that performance in such tasks is influenced, and even dominated (for large perturbations) by explicit re-aiming strategies rather than implicit adaptation.^21,22,63–69^ As such, we focused on studies that included an aftereffect phase in which the perturbation was removed.

**Figure 3:**
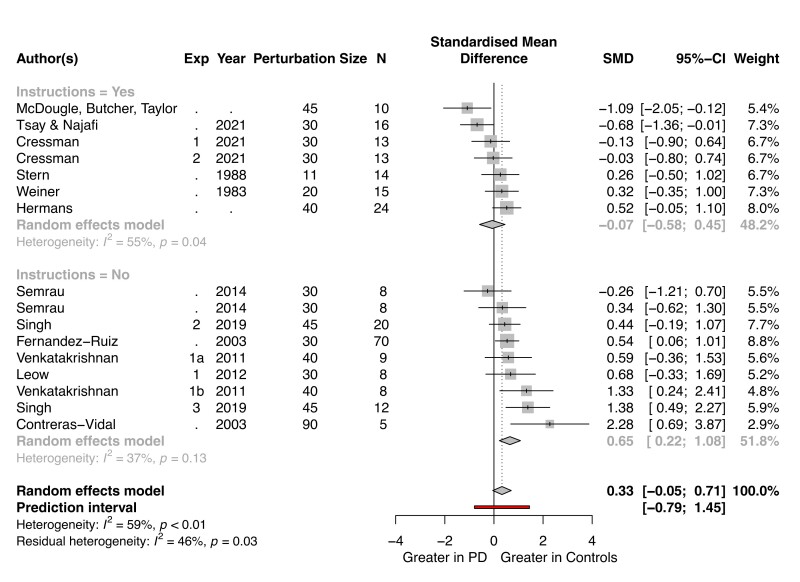
**Implicit adaptation is preserved in Parkinson's disease over a wide range of studies.** The data for this meta-analysis are taken from 16 experiments reported in 12 papers. Studies are organized according to whether the instructions at the start of the washout block explicitly emphasized that participants should stop using a strategy (top) or were ambiguous (bottom). All participants were tested on their regular medical schedule and (when applicable) with DBS turned on. The grand effect size, along with the 95% CI, across all of the experiments is indicated at the bottom of this forest plot (diamonds).

A summary of the meta-analysis is presented in Fig. 3. Positive effect sizes indicate results in which the aftereffect measure was larger for the control group compared with Parkinson's disease group (i.e. impaired adaptation in Parkinson's disease); negative values indicate a greater aftereffect for Parkinson's disease group. When the confidence interval for a given study includes 0, the group comparison was not significant. This null pattern holds in 12 of the 16 experiments, and the grand effect size also encompasses 0 [*D* = 0.33 ( − 0.05, 0.71)]. Thus, the overall pattern in the meta-analysis indicates that implicit adaptation is not affected by Parkinson's disease.

Note that we opted to only include studies examining Parkinson's disease participants on their normal medication regimen to (i) control for medication schedule and (ii) avoid double dipping, that is, counting the same individual twice—on and off medication− in the same meta-analysis (see:^19,70,71^). Nonetheless, there were three visuomotor adaptation studies in which aftereffect data were available for Parkinson's disease participants who had been tested off medication. The experiments reported in Singh *et al*. (2019) found attenuated aftereffects in Parkinson's disease tested off medication [*D* = 2.61 (1.77, 3.46)] or off DBS [*D* = 2.20 (0.27, 3.22)]. However, the results from the other two studies indicate null effects [Cressman *et al*. (2021), Exp 1: *D* = − 0.48 ( − 1.26, 0.30); Exp 2: *D* = 0.23 ( − 0.53, 1.01); Semrau *et al*. (2014), clockwise rotation, off-med: *D* = − 1.49 ( − 2.57, − 0.41); counterclockwise rotation, off-med: *D* = 0.30 (−0.66, 1.26)]. Although the sample size here is quite limited, these results suggest that implicit adaptation remains intact in individuals with Parkinson's disease even when tested outside their normal medical regimen.

We recognize that aftereffect measures may not always provide a clean measure of implicit adaptation. For example, if participants are not explicitly told to reach directly to the target, terminating the use of any strategy they may have adopted, aftereffect performance may measure both implicit adaptation and residual strategy use.^61,65,72^ The significant heterogeneity ($I^{2}=\;59\text{\%},\;P<0.01$) in the 16 experiments may, in part, be driven by differences in the instructions provided prior to the washout block. To focus on studies providing the ‘purest’ measure of implicit adaptation, we added a stricter inclusion criterion in a secondary analysis, requiring that the participants had been instructed to stop using a strategy and reach directly to the target during the aftereffect trials. Seven experiments remained after we imposed this additional criterion (top half of Fig. 3). Six of the seven studies observed no group difference, and the other showed greater implicit adaptation in Parkinson's disease. The grand effect size comparing Parkinson's disease and control groups again encompassed 0 [*D* = − 0.07 ( − 0.58, 0.45)]. Together, this subgroup analysis implies that implicit adaptation, when cleanly isolated, is not impaired in Parkinson's disease.

The studies that did not clearly instruct participants to stop using their strategy are shown in the bottom portion of Fig. 3. Here, there was a group difference [*D* = 0.65 (0.22, 1.08)], with the control participants exhibiting a larger aftereffect than Parkinson's disease participants. Notably, Parkinson's disease participants in most of these studies also showed attenuated performance during the trials right before the aftereffect block (i.e. late adaptation), a phase in which the use of an explicit re-aiming strategy is encouraged. As such, a parsimonious interpretation of these data is that the late adaptation deficit is due to impairment in strategic aiming, and the corresponding aftereffect deficit is due to greater residual strategy use in the control participants.

Previous studies have shown that, while re-aiming strategies scale with the size of the rotation, implicit adaptation remains relatively invariant.^73,74^ That is, participants explicitly re-aim more to compensate for a 90° rotation compared with a 45° rotation, but the extent of implicit adaptation is similar across a large range of perturbation sizes.^28^ This leads to an interesting prediction involving ‘contaminated’ aftereffect measures (that is studies, that do not provide instructions to stop aiming and reach directly to the target): If Parkinson's disease selectively impacts strategy use, their impairment should increase with the size of the rotation since larger rotations impose greater demands on strategy use. Although the number of data points is small, the pattern is consistent with this prediction (Supplementary Figure 2, *r*_*s*_ = 0.65, *P* = 0.06). In contrast, this relationship was not observed in the studies that used instructions designed to eliminate aiming from the aftereffect data (*r*_*s*_ = −0.17, *P* = 0.69). In summary, the meta-analysis results from the instruction-based subgroups points to a dissociation whereby Parkinson's disease does not impact implicit adaptation but does impair more explicit aspects of performance on visuomotor adaptation tasks.

## Discussion

The basal ganglia are an integral part of motor system, contributing to the acquisition and automatization of skilled movements. Studies involving individuals with Parkinson’s disease have reinforced this notion, showing deficits in a wide range of motor learning tasks.^5–11^ Here, we homed in on a critical component of sensorimotor learning, evaluating the integrity of implicit adaptation in individuals with Parkinson's disease. Although this topic has been addressed in many studies, an assortment of methodological issues has precluded a clear answer on the basic question of whether implicit adaptation is disrupted in Parkinson's disease.

In revisiting this question, we used non-contingent visual feedback in a visuomotor adaptation task, a method in which performance changes are completely implicit; as such, this method isolates implicit motor adaptation without the influence of cognitive strategies.^24^ Using both variable (Exp 1) and fixed (Exp 2) perturbations, we found that the form and extent of implicit adaptation was similar between Parkinson's disease and controls. This moderately strong null effect was observed in response to a wide range of error sizes (3°–45°). We complemented our experiments with a meta-analysis, identifying 16 experiments involving over 200 Parkinson's disease participants that included a measure that presumably isolates implicit adaptation (i.e. aftereffect). The overall pattern in these studies, as well as the aggregated effect size, indicated that implicit adaptation was not affected by Parkinson's disease.

We recognize that the literature does include positive results, cases in which Parkinson's disease participants were impaired on sensorimotor adaptation tasks. Some of the positive results could reflect Type I errors, a problem that is amplified considering the substantial within-subject variability as well as relatively small sample sizes typical in most neuropsychological studies. One source of heterogeneity may be symptom severity and medication status. For example, studies yielding positive results might involve participants with more severe motor symptoms compared with our sample in which Parkinson's disease participants tended to have mild-to-moderate symptoms. We also note that we only tested Parkinson's disease participants who were following their typical medication regimen. However, we failed to find any correlation between the UPDRS score on motor dysfunction and the extent of implicit adaptation. Moreover, the literature does not point to a consistent effect of medication on adaptation: While Singh *et al*. (2019)^19^ found attenuated aftereffects in Parkinson's disease tested off medication, two other studies reported no effect of medication (Cressman *et al*. (2021); Semrau *et al*. (2014)^70,71^). Future studies with greater sample sizes may be able to directly assess whether medication and clinical factors may jointly impact the extent of adaptation in Parkinson's disease.

It is also important to consider if prior reports of impaired motor adaptation in Parkinson's disease might reflect impairment in the utilization of explicit strategic processes.^21,22^ We tried to minimize the contribution of strategic aiming by focusing our meta-analysis on the aftereffect, measured when the perturbation has been removed. However, this assumption only holds if instructions explicitly instruct the participants to stop using any strategy and aim the ‘hand’ directly to the target. Without these instructions, the aftereffect measure may also include a contribution from an aiming strategy that was operative during late adaptation (since participants were never told to stop re-aiming).^75^ As shown in Fig. 3, this concern may be relevant for over half of the studies included in our meta-analysis. If the meta-analysis is restricted to studies in which participants were explicitly instructed in the washout phase to reach directly to the target (i.e. terminate the use of an aiming strategy), we observe a null effect, providing converging evidence that implicit adaptation is preserved in Parkinson's disease.

In contrast, the meta-analysis suggests that participants with Parkinson's disease may be impaired in deriving and/or applying an aiming strategy to counteract a perturbation. Control participants exhibited a larger aftereffect when the instructions failed to instruct participants to stop using an aiming strategy. Under such conditions, explicit aiming likely contributes to the aftereffect. A finer-grain analysis of the two studies that show the largest Parkinson's disease deficit supports this hypothesis. Contreras *et al*.^18^ found that, following exposure to a 90° rotation, Parkinson's disease group exhibited a marked reduction in the magnitude of the aftereffect relative to a control group. However, the aftereffect for the control group is likely contaminated by explicit strategies: The mean was three times larger (∼60°) than that typically observed when participants are instructed to reach directly to the target during washout (∼20°).^28,73,74^ Moreover, a similar degree of impairment in Parkinson's disease group was observed during late adaptation, a phase in which the use of an explicit strategy to counteract the perturbation is essential for successful performance. A similar pattern is found in Singh *et al*. (2020) where the perturbation was a 45° rotation: Both late adaptation and aftereffects show a similar impairment in Parkinson's disease, with the control group again showing an approximate 2-fold increase in the aftereffect size compared with the typical value found in the literature when steps are taken to eliminate explicit contributions to the aftereffect. Thus, the difference between Parkinson's disease and control groups may reflect Parkinson's disease-related impairment in strategic aiming, with the control group using a more effective aiming strategy than Parkinson's disease group, rather than a Parkinson's disease-related deficit in implicit adaptation.

Consideration of other phenomena observed in studies of sensorimotor adaptation also point to a Parkinson's disease impairment in explicit strategy use. For instance, savings, a phenomenon characterized by accelerated learning upon re-exposure to a perturbation,^17,64,76–78^ is impaired in Parkinson's disease^79^ (but see:^80^). Recent studies have shown that savings arises from the faster recall of a previously learned strategy^76^; in contrast, savings are not found in measures of implicit adaptation.^27^ A related phenomenon involves tasks in which the participant must successively learn multiple visuomotor mappings (e.g. 45° clockwise rotation block followed by 45° counterclockwise rotation block). Performance on these tasks is facilitated by the successful utilization of multiple aiming strategies.^81^ Here, too, an impairment has been observed in Parkinson's disease.^82^ Moreover, learning in response large perturbations that are suddenly introduced has been shown to demand greater strategy compared with conditions in which the perturbation is introduced incrementally.^83,84^ Parkinson's disease participants were found to be impaired in the former, but not in the latter.^20^ Future studies using methods that directly probe explicit re-aiming (see:^21,65^) can directly test strategy use in Parkinson's disease.

The absence of a Parkinson's disease-related impairment in implicit sensorimotor adaptation stands in contrast to the marked impairment observed in individuals with spinocerebellar degeneration on this form of learning.^85,86^ This cerebellar-related deficit has been observed across a wide range of tasks using different perturbation sizes,^87^ perturbation schedules,^88^ perturbation types^85,89^ and effectors.^90–92^ For example, using clamped feedback, Morehead *et al*. (2017) found that implicit adaptation was attenuated by ∼50% in a group of individuals with cerebellar degeneration. Taken together, there is a clear dissociation between the effects of degenerative processes impacting the basal ganglia or cerebellum: At least at the group level, implicit adaptation is dependent on the integrity of the cerebellum and not the basal ganglia. Future neuroimaging studies paired with voxel-based morphometry analyses can provide a more fine-grained analysis to look at individual differences within each group and assess differential contributions of subregions within each structure.

It remains to be seen if the insights gleaned from the current study also call into question other lines of evidence indicating a Parkinson's disease-related impairment in implicit sensorimotor learning. Our empirical findings and the meta-analysis highlight the challenge faced when using tasks as models of specific learning processes: Namely, most tasks likely involve multiple learning processes and care must be taken to isolate the contribution of each process as well as the interaction between different processes (see also:^93^). To take one example, studies of the effect of Parkinson's disease on sequence learning have also yielded ambiguous results, with some studies reporting deficits in sequence learning in Parkinson's disease^5,8,33^ and others finding no impairment.^94–96^ Measures of sequence learning frequently involve the combined effects of implicit and explicit processes.^97,98^ Even the acquisition of simple stimulus-response contingencies or mirror drawing do not rely exclusively on implicit processes to support incremental error-based learning. Performance on these tasks can benefit from the use of higher level heuristics.^99–104^ It will be interesting to explore if the impaired performance observed in Parkinson's disease participants on tasks traditionally thought to reflect implicit learning may instead reflect an impairment in other, more explicit forms of learning. Similar to the approach taken here, it will be important to revisit these learning domains using methods or tasks that provide purer measures of implicit and explicit processes.

## Supplementary Material

fcac303_Supplementary_Data

## Data Availability

All data can be accessed at https://github.com/xiaotsay2015/PD_implicit.

## Abbreviations

MCI =: mild cognitive impairment
MoCA =: Montreal cognitive assessment
PD =: Parkinson’s disease
UPDRS =: unified Parkinson’s disease rating scale
