# Supplementary material for: Implicit sensorimotor adaptation is preserved in Parkinson’s disease
Source: Brain Commun. 2022 Nov 21;4(6):fcac303. doi: 10.1093/braincomms/fcac303 (PMC9750131; doi:10.1093/braincomms/fcac303)
Supplement: fcac303_Supplementary_Data [file fcac303_supplementary_data.pdf]

Supplemental Methods: Meta-analysis of the Effect of PD on Visuomotor Adaptation

We performed a meta-analysis of the effect of PD on visuomotor adaptation. For this analysis, we used Google Scholar to conduct searches using the keywords: "Parkinson's Disease," "visuomotor adaptation", "visuomotor rotation," and "prism adaptation". Study titles and abstracts were examined independently by three of the authors (JT, LS, TN) who applied the following inclusion criteria: 1) The study had to employ a visuomotor adaptation task (e.g., prism adaptation, visuomotor rotation); 2) To control for medication state, the PD participants needed to be tested on their normal medication regimen (this led to exclusion of three experiments in [1–3]; 3) The study had to include a dependent variable based on data from a post-perturbation phase (aftereffect). We opted to include the third criterion to focus on a measure of implicit adaptation less "contaminated" by strategy use.

The search yielded 4320 matches. Ultimately, only 12 papers, reporting the results from 16 experiments (253 total participants) met our three inclusion criteria. Of these 16 experiments, one was the Experiment 2 in our main text, and three were unpublished (also known as "gray literature"; data obtained via personal correspondence with the authors). The inclusion of unpublished work in a meta-analysis is recommended to reduce publication bias [4].

We used Cohen's D as our measure of effect size for the between-group comparison (PD vs Control). For each of the 16 experiments, we calculated the aftereffect in one of three ways. 1) When we had access to the data, we calculated the effect size directly using the data from the no-feedback washout trials (McDougle, Butcher, and Taylor's unpublished observations, Exp 1 in this manuscript); 2) When included in the publication, we used the reported effect size [2,3,5–7]. 3) When the effect size could not be directly inferred from the reported statistics but the washout data were presented graphically, we used Webplot Digitizer (https://automeris.io/WebPlotDigitizer/) to

extract the aftereffect from the relevant figure, and used those values to calculate the effect size [1,8–12].

Aftereffect measures may not always provide a clean measure of implicit adaptation. First, the magnitude of the aftereffect can be markedly influenced by the instructions, and in particular whether participants are instructed to terminate the use of an aiming strategy and reach directly towards the visual target [13]. If this instruction is not specified, some participants are likely to continue using a strategy adopted during the perturbation phase; thus, their aftereffect will include contributions from both implicit adaptation and residual strategy use [14,15]. Second, in some studies veridical feedback is provided during the aftereffect block. When provided, the aftereffect is short-lived as participants respond to the error; indeed, participants may become aware of their adapted state and employ a re-aiming strategy to move back towards the target. To focus on studies providing the purest measure of implicit adaptation, we performed a secondary analysis with a stricter inclusion criterion. Here we only included studies in which the participants were instructed at the start of the aftereffect block to stop using a strategy and reach directly to the target.

Supplemental Covariate Analysis

Experiment 1, Analyzing rotation size as a categorical variable

In the main manuscript, Rotation Size was treated as a continuous variable in Experiment 1 to estimate the function relating the size of the motor correction in response to clamp size. We reasoned that the slope of the motor correction function (Fig 1) serves as a measurement of error sensitivity. That is, a large slope signifies higher sensitivity to visual errors, whereas a small slope signifies lower sensitivity to visual errors. We did not find any group differences in the slope of this function, indicating the PD did not impact the system's sensitivity to errors.

Rotation Size can also be analyzed as a categorical variable. As expected for errors ranging in size from 3° to 45° [16], there was a main effect of rotation size ($F_{3,102} = 18.3, p < 0.001, , \eta_p^2 = 0.4$). Post-hoc comparisons revealed that implicit adaptation was greater for larger rotations (30° and 45°) compared to smaller rotations (3° and 10°). The main effect of Group ($F_{1,33} = 0.1, p = 0.81, , \eta_p^2 = 0.0, BF_{01} = 0.2$) and the interaction between Group and Rotation Size ($F_{3,102} = 0.5, p = 0.68, , \eta_p^2 = 0.0, BF_{01} = 0.0$) were not significant. Thus, the categorical analysis also points to preserved implicit adaptation in PD.

Experiment 1, Covariate analysis of the PD group

The degree of implicit adaptation was not associated with years of education ($F_{1,33} = 0.4, p = 0.53, \beta = -0.05,\ [-0.2, 0.1], \eta_p^2 = 0.0$), MoCA score (slope computed with all rotation sizes vs

MoCA: $R = 0.1, p = 0.66, [-0.4, 0.6]$), or UPDRS score (slope with all errors vs UPDRS: $R = 0.1, p = 0.79, [-0.4, 0.5]$).

Experiment 2, Covariate analysis of the PD group

Implicit adaptation was not associated with years of education (Early: $F_{1,29} = 0.1, p = 0.74, \beta = 0.1, [-0.6, 0.8], \eta_p^2 = 0.0$; Late: $F_{1,29} = 0.6, p = 0.43, \beta = -0.7, [-2.3, 1.0], \eta_p^2 = 0.0$; Aftereffect: : $F_{1,29} = 1.7, p = 0.20, \beta = -0.9, [-2.3, 0.5]$), MoCA score (Early: $R = -0.1, p = 1, [-0.4, 0.3]$; Late: $R = 0, p = 1, [-0.3, 0.4]$; Aftereffect: $R = 0.39, p = 0.46, [-0.2, 0.5]$), or UPDRS score (Early: $R = -0.1, p = 1, [-0.4, 0.3]$, Late: $R = -0.2, p = 0.75, [-0.5, 0.2]$; Aftereffect: $R = -0.39, p = 0.12, [-0.6, 0.0]$).

Supplemental Discussion: The convergence between small and large visual errors in implicit sensorimotor adaptation.

Characterizing implicit adaptation's sensitivity to visual errors has been a contentious area of debate. One prominent hypothesis states that error sensitivity decreases with error size [17]: The system discounts large errors given that they are likely driven by rare environmental events and thus may not reflect a miscalibration in the sensorimotor system. However, this hypothesis cannot readily account for recent findings showing that implicit adaptation saturates for errors ranging from 5° to 95° [16,18]. We have proposed an alternative model (available on BioRxiv[19]) in which implicit adaptation is driven by a proprioceptive error, one that arises when the perceived hand position is biased in the direction of the cursor. Adaptation operates to nullify this proprioceptive bias. The proprioceptive bias resulting from perturbed cursor feedback saturates for large visuo-proprioceptive discrepancies[20,21]; as such, the model readily accommodates the finding that the extent of implicit adaptation will saturate over a wide range of visual errors. Given that Parkinson's disease does not impact implicit adaptation, we would hypothesize that proprioceptive re-alignment may not depend on the basal ganglia.

Supplemental Tables

Exp 1, Parkinson's Disease (n = 18)							Exp 1, Control (n = 18)			
**E1_ID**	**Age**	**Sex**	**Hand**	**MoCA**	**Education**	**UPDRS**	**Age**	**Sex**	**Hand**	**Education**
1[#]	72	M	R	27	21	16	71	M	R	16
2[#]	69	M	R	30	16	12	70	M	R	16
3[#]	81	M	R	25	27	21	78	M	R	18
4[#]	58	F	L	26	18	9	58	F	L	16
5[#]	70	F	R	28	18	11	67	F	R	16
6	65	M	R	30	16	21	65	M	R	20
7[#]	52	F	R	28	16	21	54	F	R	18
8	67	F	R	30	18	16	64	F	R	12
9[#]	64	M	R	27	20	25	64	M	R	16
10	58	F	R	30	18	20	56	F	R	12
11	76	M	R	30	18	21	74	M	R	16
12	53	M	L	29	12	21	52	M	L	16
13	63	F	A	25	14	12	62	F	A	13
14	73	F	R	28	19	10	69	F	R	14
15	66	F	R	28	16	16	64	F	R	12
16[#]	64	M	R	27	14	20	61	M	R	14
17[#]	70	F	L	30	16	10	67	F	L	14
18	61	F	R	30	16	-	58	F	R	16
**Mean<br>(SD)**	**65.7<br>(7.6)**	**8 M<br>10 F**	**14 R<br>3L, 1 A**	**27.8<br>(1.8)**	**17.4<br>(3.3)**	**16.6<br>(5.1)**	**64.1<br>(6.9)**	**8 M<br>10 F**	**14 R<br>3L, 1 A**	**15.3<br>(2.2)**
**Exp 2, Parkinson's Disease (n = 16)**							**Exp 2, Control (n = 16)**			
1[#]	71	M	R	27	21	16	72	M	R	16
2[#]	63	M	R	27	14	20	64	M	R	13
3	59	M	R	30	18	10	60	M	R	14
4[#]	69	F	L	30	16	10	64	F	L	16
5	66	M	R	25	18	21	62	M	R	16
6[#]	68	M	R	30	16	12	68	M	R	16
7[#]	80	M	R	25	27	21	78	M	R	18
8[#]	57	F	L	26	18	9	57	F	L	16
9[#]	69	F	R	28	18	11	68	F	R	14
10[#]	64	M	R	30	16	21	61	M	R	14
11	68	F	R	25	16	15	68	F	R	16
12[#]	51	F	R	28	16	21	48	F	R	16
13	73	F	R	28	20	25	73	F	R	12
14	77	F	L	29	13	18	70	F	L	23
15	69	F	R	29	16	21	72	F	R	14
16	66	F	R	30	18	16	63	F	R	12
**Mean<br>(SD)**	**66.9<br>(7.2)**	**7 M<br>9 F**	**13 R<br>3 L**	**27.4<br>(1.9)**	**17.6<br>(3.2)**	**16.7<br>(5.0)**	**65.6<br>(7.2)**	**7 M<br>9 F**	**13 R<br>3 L**	**15.4<br>(2.6)**

Supplementary Table 1: Demographic information for the PD participants and their matched control participants. Also listed are the MoCA and UPDRS scores for the PD participants.

		Early adaptation		Late adaptation		Aftereffect	
		**F-value**	**P-value**	**F-value**	**P-value**	**F-value**	**P-value**
**Main effect**	Target location	0.2	0.68	0.7	0.25	0.9	0.36
	Group	0.4	0.54	0.2	0.63	1.1	0.30
	Clamp size	1.8	0.19	1.4	0.25	3.0	0.09
**Interaction**	Target location x Group	0.3	0.61	0.2	0.65	1.0	0.32
	Target location x Clamp size	0.0	0.96	0.3	0.61	1.2	0.29
	Group x Clamp size	0.3	0.57	0.2	0.69	0.0	0.88
	Group x Clamp size x Target location	0.3	0.61	0.0	0.95	0.0	0.93

Supplementary Table 2: Parkinson's Disease does not impact learning functions in response to clamped feedback at both target locations. None of the main effects and interactions were significant.

Supplemental Figures

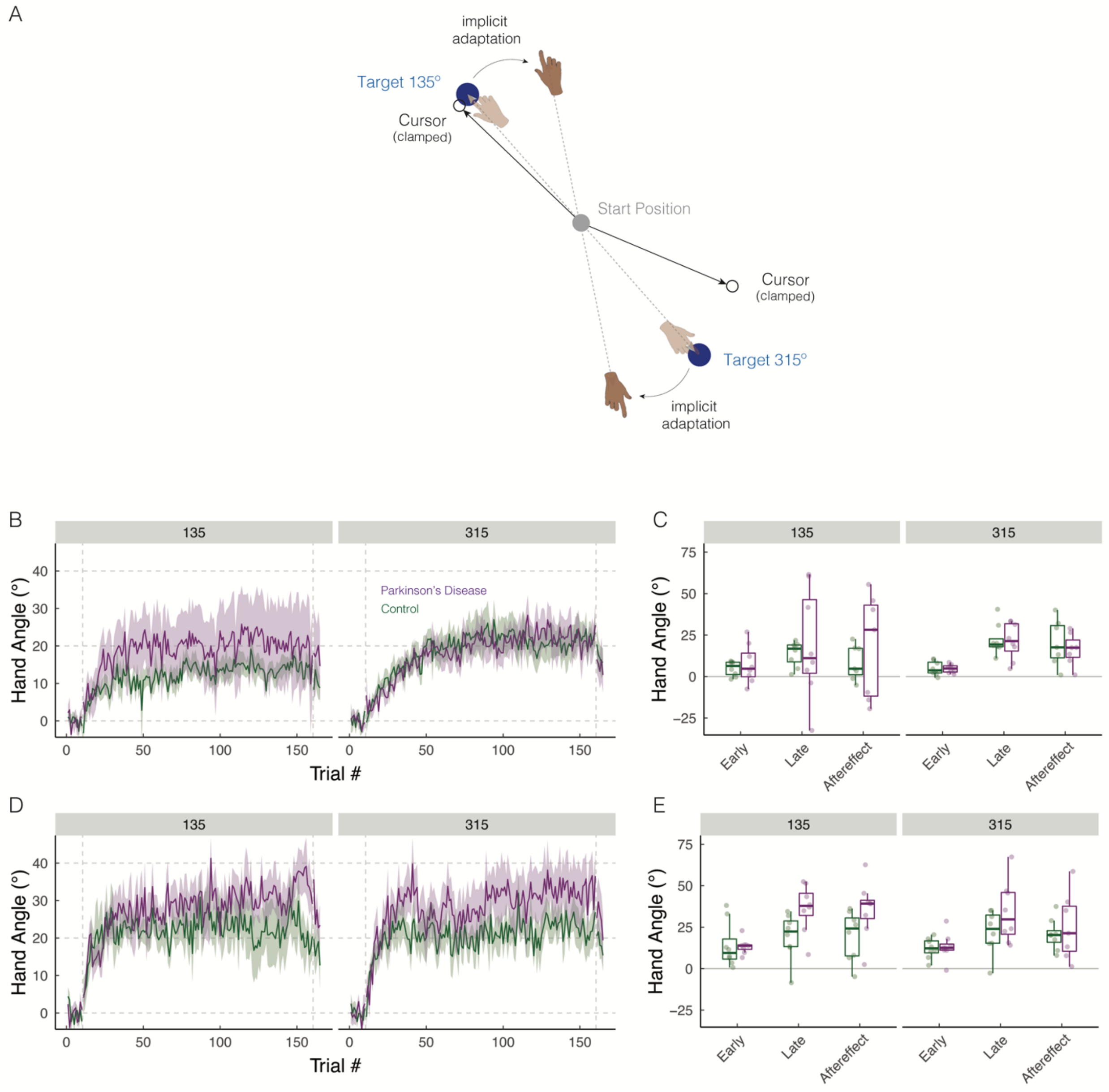

Supplementary Figure 1: Parkinson's Disease does not impact learning functions in response to clamped feedback at both target locations. **(a)** Schematic of the clamped feedback task at two target locations in Experiment 2. The cursor feedback (hollow black circle) followed an invariant trajectory, rotated by either 3° or 30° relative to either the 135° target (top-left) or the 315° target (bottom-right). Participants were instructed to always move directly to the target (blue circle) and ignore the visual feedback. The translucent and solid colors display hand position early and late in adaptation, respectively. One clamp size was used for a block of 110 reaches to one of the locations. The other clamp size was used for a second block of 110 reaches, now directed to the other location. The assignment of clamp size and direction to target locations was counterbalanced across participants. Clamp direction was the same in both perturbation blocks. **(b)** Mean time course of hand angle for the 3° condition for the PD (dark magenta) and

Control (green) participants, with the panels corresponding to the 135° and 315° target locations. Data for each participant were baseline adjusted, by subtracting the mean hand angle during the baseline phase with veridical feedback. Shaded region denotes SEM. **(c)** Average hand angle during early and late phases of the perturbation block, and during the no-feedback aftereffect block for the 3° condition. Box plots denote the median (thick horizontal lines), quartiles (1st and 3rd, the edges of the boxes), and extrema (min and max, vertical thin lines). The mean for each participant is shown as translucent circles. None of the group comparisons between PD and Controls were significant. **(d, e)** Same as **(b,c)**, but for the 30° condition.

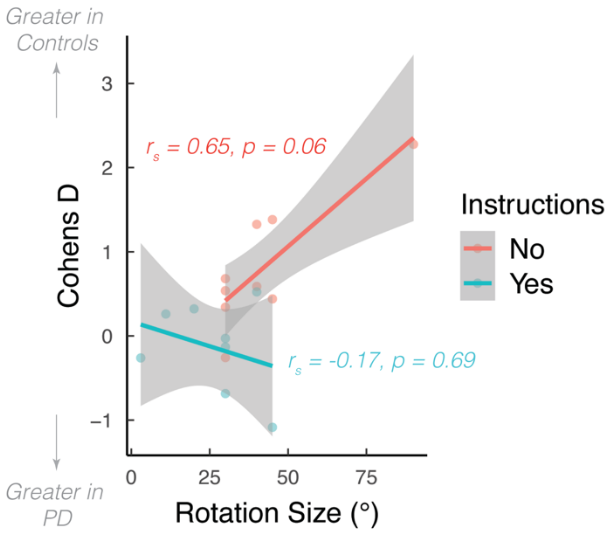

Supplementary Figure 2: Performance difference between PD and control participants is observed in response to perturbations that engage aiming strategies. Blue dots indicate studies in which participants were instructed to not aim; red dots indicate studies in which no instructions were given and, presumably, aiming strategies would be more prevalent as rotation size increased.

Supplemental References

1. Singh P, Lenka A, Stezin A, et al. Basal ganglia contributions during the learning of a visuomotor rotation: Effect of dopamine, deep brain stimulation and reinforcement. *Eur J Neurosci*. 2019;50(8):3349-3364.

2. Semrau JA, Perlmutter JS, Thoroughman KA. Visuomotor adaptation in Parkinson's disease: effects of perturbation type and medication state. *J Neurophysiol*. 2014;111(12):2675-2687.

3. Cressman EK, Salomonczyk D, Constantin A, et al. Proprioceptive recalibration following implicit visuomotor adaptation is preserved in Parkinson's disease. *Exp Brain Res*. Published online March 10, 2021. doi:10.1007/s00221-021-06075-y

4. Paez A. Gray literature: An important resource in systematic reviews. *J Evid Based Med*. 2017;10(3):233-240.

5. Leow LA, Marinovic W, de Rugy A, Carroll TJ. Task errors contribute to implicit aftereffects in sensorimotor adaptation. *Eur J Neurosci*. 2018;48(11):3397-3409.

6. Contreras-Vidal JL, Buch ER. Effects of Parkinson's disease on visuomotor adaptation. *Exp Brain Res*. 2003;150(1):25-32.

7. Mongeon D, Blanchet P, Messier J. Impact of Parkinson's disease and dopaminergic medication on adaptation to explicit and implicit visuomotor perturbations. *Brain Cogn*. 2013;81(2):271-282.

8. Hermans P, Broeder S, Vandendoorent B, Nieuwboer A, de Xivry JJO. Motor adaptation processes decline with aging, but not with Parkinson's disease. In: ; 2021.

9. Weiner MJ, Hallett M, Funkenstein HH. Adaptation to lateral displacement of vision in patients with lesions of the central nervous system. *Neurology*. 1983;33(6):766-772.

10. Venkatakrishnan A, Banquet JP, Burnod Y, Contreras-vidal JL. Parkinson's disease differentially affects adaptation to gradual as compared to sudden visuomotor distortions. *Hum Mov Sci*. 2011;30(4):760-769.

11. Gutierrez-Garralda JM, Moreno-Briseño P, Boll MC, et al. The effect of Parkinson's disease and Huntington's disease on human visuomotor learning. *Eur J Neurosci*. 2013;38(6):2933-2940.

12. Stern Y, Mayeux R, Hermann A, Rosen J. Prism adaptation in Parkinson's disease. *J Neurol Neurosurg Psychiatry*. 1988;51(12):1584-1587.

13. Morehead R, de Xivry JJO. A Synthesis of the Many Errors and Learning Processes of Visuomotor Adaptation. *BioRxiv*. Published online March 15, 2021. doi:10.1101/2021.03.14.435278

14. Werner S, van Aken BC, Hulst T, et al. Awareness of sensorimotor adaptation to visual rotations of different size. *PLoS One*. 2015;10(4):e0123321.

15. Maresch J, Werner S, Donchin O. Methods matter: Your measures of explicit and implicit processes in visuomotor adaptation affect your results. *Eur J Neurosci*. 2020;(ejn.14945). doi:10.1111/ejn.14945

16. Kim HE, Morehead R, Parvin DE, Moazzezi R, Ivry RB. Invariant errors reveal limitations in motor correction rather than constraints on error sensitivity. *Commun Biol*. 2018;1:19.

17. Wei K, Körding K. Relevance of error: what drives motor adaptation? *J Neurophysiol*. 2009;101(2):655-664.

18. Morehead R, Taylor JA, Parvin DE, Ivry RB. Characteristics of Implicit Sensorimotor Adaptation Revealed by Task-irrelevant Clamped Feedback. *J Cogn Neurosci*. 2017;29(6):1061-1074.

19. Tsay JS, Kim HE, Haith AM, Ivry RB. Proprioceptive Re-alignment drives Implicit Sensorimotor Adaptation. *bioRxiv*. Published online December 23, 2021. doi:10.1101/2021.12.21.473747

20. Ruttle JE, Hart BM ’t, Henriques DYP. Implicit motor learning within three trials. *Sci Rep*. 2021;11(1):1627.

21. ’t Hart BM, Ruttle JE, Henriques DYP. Proprioceptive recalibration generalizes relative to hand position. Published October 26, 2020. Accessed December 29, 2020. https://deniseh.lab.yorku.ca/files/2020/05/tHart_SfN_2019.pdf?x64373
